# Editorial: The Network of Inflammatory Mechanisms in Kidney Disease: Mechanism and New Therapeutic Agents

**DOI:** 10.3389/fmed.2021.799850

**Published:** 2021-12-09

**Authors:** Yuji Nozaki, Poh-Yi Gan, Joshua Daniel Ooi

**Affiliations:** ^1^Department of Hematology and Rheumatology, Kindai University Faculty of Medicine, Osaka, Japan; ^2^Centre for Inflammatory Disease, Department of Medicine, Monash Medical Centre, School of Clinical Sciences, Monash University, Clayton, VIC, Australia; ^3^Department of Immunology, Monash Health, Monash Medical Centre, Clayton, VIC, Australia

**Keywords:** therapeutic agents, kidney disease, autoimmune disease, cytokines, signaling pathways

It has been shown that the progression of kidney disease in humans and animal models usually involves the sustained activation of a number of signaling pathways. In this research theme, immunological mechanisms and genetic analysis that are important in the pathogenesis of various renal diseases were studied, and new perspectives on renal diseases were discussed. This review presents an overview of the papers submitted to this Research Topic.

In autoimmune glomerulonephritis, Nagai reviewed the signaling mechanisms and functional roles of typical T-cell co-inhibitory receptors in the control of autoimmune glomerulonephritis. In models of autoimmune vasculitis affecting the kidney, pathogenesis is characterized by early Th17 dominance followed by Th1 dominance and reduced Treg cell function of autoreactive T cells. Thus, further elucidation of the suppressive effects and pathways in immune kidney disease is needed to develop effective therapies for T cell-mediated autoimmune glomerulonephritis.

Two interesting papers on the pathogenesis of renal fibrosis have been submitted to this Research Topic. Hirooka et al. demonstrated in a mouse model of renal fibrosis that interleukin (IL)-18, a cytokine important for the induction of Th1 responses, plays an important role in renal interstitial fibrosis. Furthermore, Foxp-3 positive cells and regulatory T cells were found to be effective in preventing fibrosis in the renal interstitium through the IL-18 receptor signaling pathway.

Xu et al. showed that cilomilast, a second-generation selective phosphodiesterase-4 inhibitor, inhibited the activation of fibroblasts stimulated by TGF-β1 and reduced the expression of fibronectin, α-SMA, collagen I, and collagen III expression. In addition, cilomilast inhibited the activation of TGF-β1-Smad2/3 signaling in fibroblasts stimulated by TGF-β1.

Yang et al. reported the nephroprotective effect of erythropoietin (EPO) on contrast-induced nephropathy in a mouse model. *In vitro*, EPO was shown to increase protein levels of Janus kinase 2/signal transducer and activator of transcription 3 (JAK2/STAT3) signaling pathway components and inhibit apoptosis.

In a mouse model of septic acute kidney injury, Wang et al. described the protective effect of heparin against septic acute kidney injury via neutralization of extracellular histones. Based on a variety of perspective, it is suggested that heparin may play a protective role by neutralizing histones, thereby mitigating apoptosis and inflammation.

Gao et al. analyzed the efficacy and safety of rituximab, one of the first-line drugs for intermediate and high-risk primary membranous nephropathy, in 95 patients during a 6-month observation period. Results of multivariate logistic regression analysis showed that severe proteinuria and persistent anti-PLA2R antibody positivity were independent risk factors for non-remission. The remission rate with rituximab as first-line therapy was higher than that with alternative agents, and only one case of discontinuation due to adverse events was reported, indicating the efficacy and safety of rituximab as first line therapy.

Liu et al. investigated the risk factors for treatment failure in 241 patients with peritonitis, a serious complication of peritoneal dialysis (PD). After logistic regression analysis, they reported that fibrinogen, PD duration, fungal infection, history of hemodialysis, concomitant intestinal obstruction, and diabetes were independent risk factors for poor peritonitis outcome, while high-density lipoprotein was a protective factor.

Selvaraja et al. reported that HLA-DRB1^*^0405, HLA-DRB1^*^1502, and HLA-DRB1^*^1602 were associated with increased risk of developing systemic lupus erythematosus (SLE). HLA-DRB1^*^0405, HLA-DRB1^*^1502, and HLA-DRB1^*^1602 were associated with an increased risk of developing SLE, while HLA-DRB1^*^1201 and HLADRB1^*^1202 were associated with a decreased risk of developing SLE. In addition, HLA-DRB1^*^04 was significantly associated with lupus nephritis and arthritis, and HLA-DRB1^*^15 with stomatitis. Analysis of the association between HLA-DRB1^*^04 and clinical and biological factors showed that HLA-DRB1^*^04 was significantly associated with SLE disease activity index score, antinuclear antibodies, C-reactive protein in blood, and urinary protein. SLE carriers with the HLA-DRB1^*^04 allele were significantly associated with elevated levels of pro-inflammatory cytokines compared to SLE carriers without the HLA-DRB1^*^04 allele. Based on these results, the authors indicate that the disease severity of SLE may be determined by the HLA-DRB1 allele.

Overall, this Research Topic introduces the immunological mechanism of action from basic research and the efficacy of drugs and genetic analysis for renal diseases from clinical practice to better understand the new inflammatory mechanisms in renal diseases. Although renal diseases are commonly experienced in clinical practice, autoimmune mechanisms play a variety of roles, the details of which have yet to be elucidated. We hope that basic and clinical researchers will present and evaluate each other's research results through the Research Topics to further elucidate the pathogenesis in this field.

## Author Contributions

All authors listed have made a substantial, direct, and intellectual contribution to the work and approved it for publication.

## Funding

JO was supported by an Al and Val Rosenstrauss Fellowship by the Rebecca Cooper Medical Research Foundation.

## Conflict of Interest

The authors declare that the research was conducted in the absence of any commercial or financial relationships that could be construed as a potential conflict of interest.

## Publisher's Note

All claims expressed in this article are solely those of the authors and do not necessarily represent those of their affiliated organizations, or those of the publisher, the editors and the reviewers. Any product that may be evaluated in this article, or claim that may be made by its manufacturer, is not guaranteed or endorsed by the publisher.

